# Enhanced 2-keto-l-gulonic acid production by applying l-sorbose-tolerant helper strain in the co-culture system

**DOI:** 10.1186/s13568-018-0562-y

**Published:** 2018-02-28

**Authors:** Mandlaa, Ziyu Sun, Ruigang Wang, Xiaodong Han, Hui Xu, Weichao Yang

**Affiliations:** 1 College of Food Science and Engineering, Inner Mongolia Agricultural University, 010018 Hohhot, China; 2 College of Life Science, Inner Mongolia Agricultural University, 010018 Hohhot, China; 3 Institute of Applied Ecology, Chinese Academy of Sciences, 72 Wenhua Road, 110016 Shenyang, China

**Keywords:** 2-Keto-gulonic acid, Co-culture system, Helper strain, l-Sorbose, Vitamin C

## Abstract

2-Keto-l-gulonic acid (the precursor of vitamin C) is bio-converted from l-sorbose by mixed fermentation of *Ketogulonicigenium vulgare* and a helper strain. The helper strain promotes the conversion of 2-KLG by enhancing the growth of *K. vulgare*, but its growth is greatly inhibited by high concentration of l-sorbose, which consequently influence the 2-KLG production. The aim of this study is to obtain l-sorbose-tolerant helper strain (LHS) by experimental evolution for reduced l-sorbose-inhibition-effect and enhanced 2-KLG productivity in high concentration of l-sorbose. After three steps screening by using our devised screening strategy, three strains (i.e., Bc 21, Bc 47, Bc 50) with high resistance to high concentration of l-sorbose were obtained. The fermentation tests by co-culturing Bc 21 and *K. vulgare* 418 showed that the production of 2-KLG was increased by 17.9% in 11% l-sorbose medium than that in 8% after 55 h of fermentation and the conversion rate was 89.5%. The results suggested that Bc 21 could be an ideal helper strain for 2-KLG production under high concentration of l-sorbose and demonstrated the feasibility of using experimental evolution to breed LHS for vitamin C production.

## Introduction

2-Keto-l-gulonic acid (2-KLG) is a precursor of vitamin C (Bremus et al. 2006), and it is mainly bio-converted from l-sorbose via a co-culture system in two-step fermentation process of vitamin C (Pappenberger and Hohmann 2014). This co-culture system is composed by two strains: *Ketogulonicigenium vulgare* (previously identified as *Gluconobacter oxydans*) (Urbance et al. 2001; Yin et al. 1980) and the helper strain (mostly belongs to *Bacillus* spp.) (Bremus et al. 2006). *K. vulgare* has the ability for converting l-sorbose to 2-KLG (Liu et al. 2011a), but its growth is very weak and the yield of 2-KLG is very low when it is cultured without the helper strain (Yin et al. 1980).

The studies on the relationship between *K. vulgare* and the helper strain have always been one of hotspots in the field of vitamin C fermentation. In recent years, with the development and application of the omics technology (such as proteomics, metabonomics, and genomics) (Ma et al. 2011, 2012; Pappenberger and Hohmann 2014; Jia et al. 2017), the mechanism of the interaction between the two bacteria has been well explained (Jia et al. 2016a, b, 2017). Most of the related studies have proved that the harmonious proportion between *K. vulgare* and helper strain in the co-culture system is a key factor for 2-KLG production with higher efficiency (Zhang et al. 1988; Xu et al. 2004; Yang et al. 2013). Moreover, in the co-culture system, l-sorbose is not only the substrate for 2-KLG production, but also plays an important role in regulating the proportion of the two strains by promoting the growth of *K. vulgare* and inhibiting the growth of helper strain (Mandlaa et al. 2015). Therefore, the inhibition effect of l-sorbose on the helper strain’s growth is vital for keeping an optimal proportion of *K. vulgare* and helper strain in 2-KLG fermentation.

Nowadays, during the industrial production of vitamin C, the proportion between *K. vulgare* and helper strain is in a harmonious status by practical experience in batch fermentation with lower L-sorbose concentration (below 8%) (Zhang et al. 1988; Xu et al. 2004; Yang et al. 2013; Zou et al. 2013). The variation of the helper strain number or l-sorbose concentration in the co-culture system will destroy this harmonious status and further lead the decrease of 2-KLG yield. Previous studies showed that addition of extra helper strain in higher l-sorbose fermentation could increase 2-KLG production by the co-culture system (Mandlaa et al. 2015). However, the method of adding extra helper strain into the co-culture system will increase the risks of contamination and production costs.

Therefore, in the present study, the l-sorbose-tolerance helper strains (LHS) were screened out by using experimental evolution strategy. The production of 2-KLG in high l-sorbose concentration was investigated by co-culture of LHS and *K. vulgare.* These results helped us obtain effective LHS and improve the 2-KLG production under high concentration of l-sorbose.

## Materials and methods

### Microbial strains and media

Two strains, *Ketogulonicigenium vulgare* 418 (2-KLG-producing strain) and *Bacillus cereus* 112 (helper strain*),* were kindly provided by Northeast Pharmaceut Group Co. Ltd (37 Zhonggong North Street, Shenyang city, China) for research and stored in Institute of Applied Ecology, Chinese Academy of Sciences.

Seed culture medium (g L^−1^): l-sorbose 20, corn-steep liquor 5, carbamide 1, CaCO_3_ 2. Fermentation medium (g L^−1^): l-sorbose 80, corn-steep liquor 10, carbamide 12, KH_2_PO_4_ 1, MgSO_4_·7H_2_O 0.2, CaCO_3_ 1 and pH 6.7–7.0 (l-sorbose and carbamide are sterilized separately). Isolation medium (g L^−1^): l-sorbose 20, yeast extraction 3, corn-steep liquor 3, carbamide 1, KH_2_PO_4_ 1, MgSO_4_·7H_2_O 0.3, CaCO_3_ 1, agar 2 and pH 6.7. The pH was adjusted with NaOH solution (40%, w/v) before sterilization.

### Screening of LHS by experimental evolution

Firstly, the tolerance of the helper strains to l-sorbose was investigated by cultivating strains in a 20 mL medium containing l-sorbose at different concentration from 10 to 50% in 250 mL flasks. After 12 h of cultivation, the OD of the helper strain was determined and the concentration of l-sorbose in medium at which the helper strain can’t grow in 12 h of cultivation was found out. Secondly, the colonies of the helper strain (had been cultivated in isolation medium for 24 h) were inoculated to a 250 mL flask containing 20 mL fermentation medium (10% of l-sorbose) and then cultivated at 29 °C and 180 rpm. After 12 h of cultivation, the fermentation broth was transferred to another 250 mL flask that containing 2.0 g l-sorbose for further 12 h of cultivation at 29 °C and 180 rpm. As above procedure, the fermentation broth was transferred continuously to a new 250 mL flask containing 2.0 g l-sorbose until the concentration of l-sorbose in the fermentation broth was 15%. Finally, the fermentation broth was centrifuged at 800×*g* at room temperature for 10 min and the supernatant was discarded. The cells of the helper strain were suspended and diluted by sterilized water and spread onto petri dishes containing 20 mL of isolation medium. After cultivation at 29 °C for 12 h, the helper strains with the biggest colonies on each petri dish were selected and inoculated into 250 mL flasks containing 20 mL fermentation medium (with 15% of l-sorbose). After 12 h of cultivation at 29 °C and 180 rpm, the ODs of the helper strains were determined, and those helper strains with more than 0.08 OD value were selected (Step 1).

In the secondary screening step, the isolated helper strains were inoculated into fermentation medium (15% l-sorbose) and cultivated in 250 mL flasks at 29 °C and 180 rpm for 12 h. The OD of the helper strains was determined at 0 and 12 h, respectively. Based on the variation of OD between 0 and 12 h, the helper strains that grew well in fermentation medium containing 15% of l-sorbose were selected to the thirdly screening step.

In the thirdly screening step, each selected helper strain was co-cultured with *K. vulgare* in two levels of l-sorbose (8 and 14%) to investigate the resistance of the selected helper strain to high concentration of l-sorbose and the 2-KLG production in co-culture fermentation system. The fermentation conditions, preparation of seeds and the proportion of inoculation were same as our previously published literature (Mandlaa et al. 2013).

### Assay methods

Determinations of 2-KLG and the optical density (at 650 nm, OD) of helper strain in the fermentation broth are according to previously published literatures, respectively (Yin et al. 1997; Liu et al. 2011b). Three replications were performed in all treatments. Differences between two groups were regarded as statistically significant if P < 0.05. Microsoft office excel 2007 was applied to analyze the data and draw the figures.

## Results

### The limited concentration of l-sorbose for the growth of helper strain

**Fig. 1:**
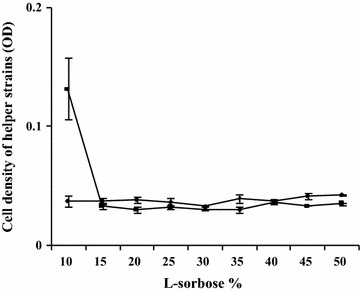
The effect of l-sorbose on the growth of helper strain. *Diamond* the OD of helper strain at 0 h of fermentation at different concentration of l-sorbose; *square* the OD of helper strain at 12 h of fermentation at different concentration of l-sorbose

A series of concentrations of l-sorbose (10–50%) were applied to evaluate the effect of l-sorbose on the growth of helper strain in 12 h of cultivation. The results were shown in Fig. 1. After 12 h of cultivation at above 15% l-sorbose concentration, the OD of helper strain showed no significant difference than that at 0 h. The result indicated that 15% l-sorbose might be a limited concentration for the growth of helper strain. Hence, this concentration (15%) of l-sorbose could be used to screen the LHS.

### Screening of LHS

**Fig. 2:**
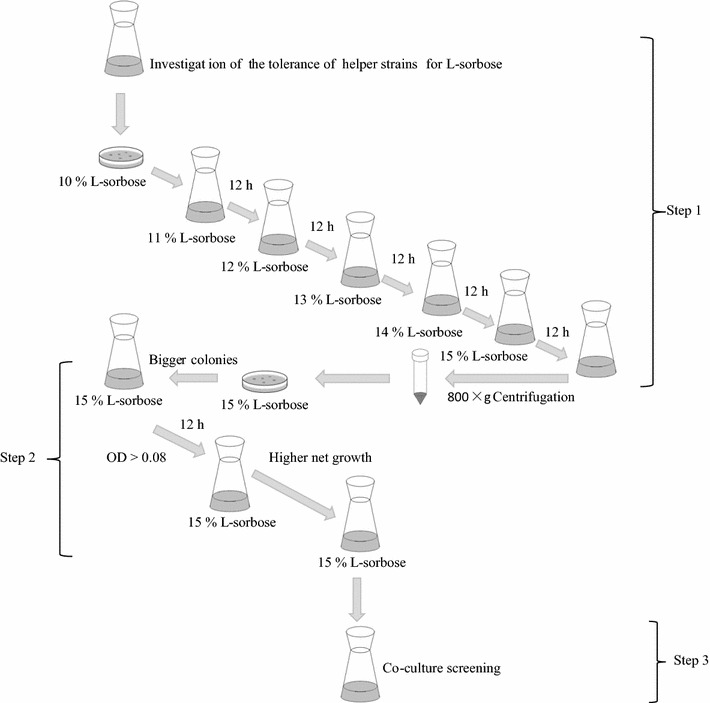
The flow-process diagram for screening l-sorbose-tolerant helper strain

**Fig. 3:**
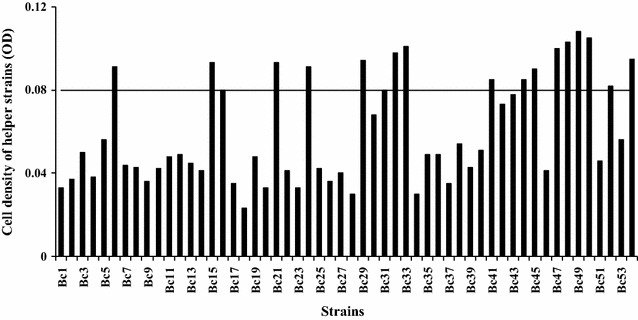
Determination of cell density of helper strains in the first screening step

**Fig. 4:**
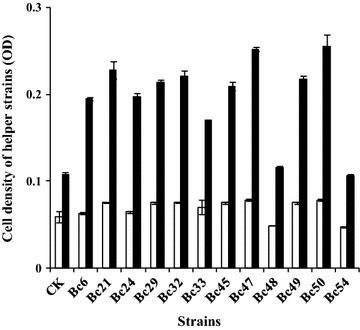
Determination of cell density of helper strains in the second screening step. Open bar: the OD of helper strain at 0 h of fermentation; filled bar: the OD of helper strain at 12 h of fermentation under the condition of 15% l-sorbose

After confirmation of the limited concentration of l-sorbose for the growth of helper strain, the screening of LHS was conducted and the screening process flow-chart was showed in Fig. 2. If the helper strain can resist the stress of higher l-sorbose concentration, its cells will be harvested by centrifugation. On the contrary, if the helper strain can’t resist the stress from l-sorbose, it might be readily to form spores or die. By using this screening strategy, 54 helper strains were selected from petri dishes after determining the size of colonies. Then they were inoculated into 250 mL flasks, respectively (Fig. 3). After 12 h of cultivation, 12 helper strains with higher OD values were selected to the second screening step. After 12 h of cultivation in 15% of l-sorbose fermentation medium, as the results showed in Fig. 4, the OD of each helper strain in 12 h was significant higher than that at 0 h. Three helper strains (Bc 21, Bc 47 and Bc 50) with a relatively higher net growth were selected and considered as the LHS to the third screening step.

### Co-culture screening of LHS and the effect of LHS on 2-KLG production

**Fig. 5:**
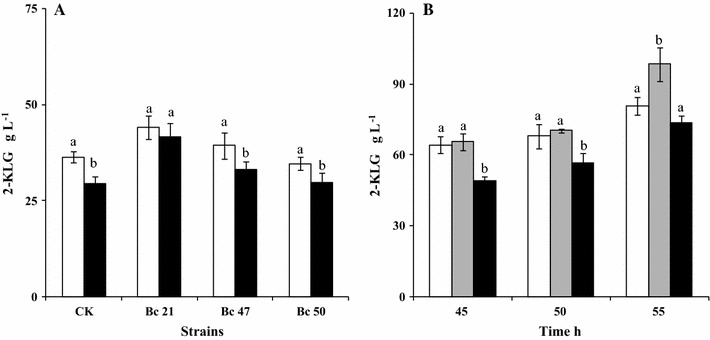
The effect of l-sorbose-tolerant helper strain on the production of 2-KLG. **A** White bar: the concentration of 2-KLG produced by co-culturing l-sorbose-tolerant helper strain and *K. vugare* at 8% of l-sorbose in the medium; black bar: the concentration of 2-KLG produced by co-culturing l-sorbose-tolerant helper strain and *K. vugare* at 14% of l-sorbose in the medium; **B** White bar: the concentration of 2-KLG in 8% l-sorbose, gray bar: the concentration of 2-KLG in 11% l-sorbose; black bar: the concentration of 2-KLG in 14% l-sorbose. The same letters (a, b and c) in the bar denote treatments are not significantly different (P > 0.05)

Three LHS were further screened by co-culturing with *K. vulgare* 418 in two concentration of l-sorbose (8 and 14%), respectively. The results were shown in Fig. 5A. After 45 h of fermentation, the 2-KLG production in both co-culture of Bc 47-*K. vulgare* 418 and Bc 50-*K. vulgare* 418 showed no significant difference with the control treatment whether in low concentration (8%) or high concentration (14%). However, the production of 2-KLG in co-culture of Bc 21-*K. vulgare* 418 was significantly higher than the control treatments in 8 or 14% l-sorbose, respectively. The results indicated the co-culture system composed by Bc 21 and *K. vulgare* 418 had better stability and adaptability whether in low (8%) or high (14%) concentration of l-sorbose.

The effects of different l-sorbose concentration on the 2-KLG production by co-culture system of Bc 21 and *K. vulgare* 418 were investigated. The results were shown in Fig. 5B. After 55 h of fermentation, the concentration of 2-KLG in 8, 11 and 14% l-sorbose medium was 83.5, 98.5 and 73.8 g L^−1^, respectively. The yield of 2-KLG was increased by 17.9% in 11% of l-sorbose medium than that in 8%, and a conversion rate of 89.5% was obtained in 11% of l-sorbose medium.

## Discussion

In the co-culture system of vitamin C, l-sorbose is not only the substrate, but also plays an important role in keeping the ratio of *K. vulgare* and the helper strain by promoting the growth of *K. vulgare* and inhibiting the growth of the helper strain (Mandlaa et al. 2013). Although the inhibition effect of l-sorbose on the growth of helper strain makes assurance of the balanced proportion of helper strain and *K. vulgare* and an effective production of 2-KLG in low concentration of l-sorbose fermentation, the inhibition effect has greatly influenced the growth of the helper strain and led a low 2-KLG production in high concentration of l-sorbose fermentation. As our results showed, the helper strain grew slowly in 10% of l-sorbose, and stopped growing when the concentration of l-sorbose was above 15%. Therefore, only raising the concentration of l-sorbose in the co-culture system will inhibit the growth of helper strain and destroy the balance of helper strain and *K*. *vulgare*. However, by adding extra helper strain to the co-culture system, the 2-KLG production can be enhanced in high concentration of l-sorbose fermentation (Mandlaa et al. 2013), which further confirmed that the growth inhibition of L-sorbose on helper strain is one of important reasons for lower 2-KLG yield in higher concentration of l-sorbose fermentation.

The experimental evolution is typically used to investigate the adaptation of microbes to specific laboratory conditions (Buckling et al. 2009; Zou et al. 2013). By using this strategy, many strains with potential application in different areas of bio-conversion were obtained (Wu et al. 2014; Harden et al. 2015; Zhang et al. 2015). In this study, an experimental evolution for 60 h of fermentation was designed to screen for the LHS. The screening strategy was based on the inhibition effect of high concentration of l-sorbose to the growth of helper strain. By gradually increasing the concentration of l-sorbose in the fermentation medium, the LHS, with enhanced resistance to high concentration of l-sorbose, could grow well and be finally screened out after a three-step screening procedure. Finally, three strains showing LHS characteristics were obtained. Compared to the original helper strain (control treatment), the screened LHS showed a stronger resistance to high concentration of l-sorbose (Fig. 4). Although it has certain fortuitousness in obtaining LHS by experimental evolution in a short time, it is enough to confirm the hypothesis that the experimental evolution strategy can be used for LHS screening. Combined with other mutagenesis breeding technology, experimental evolution can help us obtain more LHS with higher resistence to L-sorbose and higher companion effects in further study.

It was reported that osmolality was an important factor for the growth of helper strain in the co-culture system (Chen et al. 2010). At the initial stage of fermentation, high concentration of l-sorbose was the main reason for hyperosmotic stress. Moreover, the production of 2-KLG can be improved by adding sucrose (Chen et al. 2010) or glucose (Yang et al. 2008) in the co-culture system to adjust the osmotic pressure. In this study, LHS was isolated for improving the growth of helper strain under hyperosmotic stress caused by a higher concentration of l-sorbose. After a three-step screening procedure, three LHS strains (i.e., Bc 21, Bc 47, Bc 50) were obtained. According to 2-KLG yields by co-culture of LHS and *K. vulgare* in low concentration and high concentration, the co-culture of Bc 21—*K. vulgare* 418 showed the highest values and significantly higher than the control treatment. However, 2-KLG yields in both co-cultures of Bc 47-*K. vulgare* 418 and Bc 50-*K. vulgare* 418 showed no significant differences with the control treatment, respectively. The results indicated the co-culture system of Bc 21 and *K. vulgare* 418 had better stability and adaptability to high concentration of l-sorbose. This might be related to the better resistance of Bc 21 to osmotic stress than any other LHS.

According to previously studies on the hyperosmotic-resistance mechanisms of microbes, bacteria mainly resist high osmotic pressure in two ways (Beales 2004). On one way, by changing cell membrane permeability, exogenous osmolytes (such as glycine and betaine) are selectively absorbed from the environment to improve the osmotic pressure in cells. On another way, bacteria regulate their own metabolic process, and metabolize more endogenous osmolytes (such as proline and sucrose) to improve cell osmotic pressure. In the co-culture system of Bc 21 and *K. vulgare* 418, the enhanced hyperosmotic-resistance ability and increased cell number of Bc 21 in high concentration of l-sorbose might metabolized more small molecular substances, including not only the osmolytes, but also the companion substances (CSs), which were then secreted and supplied for growth of *K. vulgare* after sporulation of Bc 21 (Pappenberger and Hohmann 2014). The CSs, including amino acids, proteins and anti active oxygen factors (Ma et al. 2011, 2012), provided the necessary nutrition and reduced the persecution of active oxygen, so as to promote the growth of *K. vulgare* and further enhance the 2-KLG production.

Compared with the reported yields of 2-KLG and conversion rate, the co-culture of Bc 21 and *K. vulgare* 418 showed good fermentation capacity. Kang et al. (2008) screened out an effective helper strain-B1514, but the 2-KLG yields by B1514-*K. vulgare* was just 66.2 g L^−1^ with a low conversion rate (55.8%) in 11% l-sorbose medium after 44 h of fermentation. To our best of knowledge, the 2-KLG yields (98.5 g L^−1^) by co-culture of Bc 21-*K. vulgare* was the highest value in flask fermentation. The significantly increased 2-KLG yields (increased by 17.9%) by Bc 21 and *K. vulgare* 418 than control and the conversion rate of 89.5% in high concentration of l-sorbose (11%) indicated Bc 21 can be a candidate strain for vitamin C industrial fermentation with high concentration of l-sorbose. A higher concentration of l-sorbose not only improve the efficiency of 2-KLG production, but also reduce the probability of contamination in the co-culture system.

In conclusion, the high concentration of l-sorbose greatly inhibited the growth of the helper strain. By using the experimental evolution strategy, three l-sorbose-resistant helper strains were isolated. Of which, Bc 21 showed the most resistance ability to higher concentration of l-sorbose. After 55 h of fermentation, the co-culture system of Bc 21 and *K. vulgare* 418 increased the 2-KLG yield by 17.9% in 11% l-sorbose medium than that in 8%. These results suggest that the experimental evolution strategy can be used for LHS screening and Bc 21 is a candidate helper strain for 2-KLG production in high concentration of l-sorbose.
